# Study of the Relation between the Resonance Behavior of Thickness Shear Mode (TSM) Sensors and the Mechanical Characteristics of Biofilms

**DOI:** 10.3390/s17061395

**Published:** 2017-06-15

**Authors:** Pedro Castro, Luis Elvira, Juan Ramón Maestre, Francisco Montero de Espinosa

**Affiliations:** 1 Institute of Physical and Information Technologies, CSIC, C/Serrano, 144, 28006 Madrid, Spain; luis.elvira@csic.es (L.E.); francisco.montero@csic.es (F.M.d.E.); 2 Servicio de Microbiología Clínica, Hospital Central de la Defensa Gómez-Ulla, Glorieta del Ejército, s/n, 28047 Madrid, Spain; jmaever@oc.mde.es

**Keywords:** TSM sensor, QCM, AT-cut quartz crystal, biofilm, semi-infinite layer, mechanical characterization, shear modulus, *S. epidermidis*, *E. coli*, micro-rheological technique

## Abstract

This work analyzes some key aspects of the behavior of sensors based on piezoelectric Thickness Shear Mode (TSM) resonators to study and monitor microbial biofilms. The operation of these sensors is based on the analysis of their resonance properties (both resonance frequency and dissipation factor) that vary in contact with the analyzed sample. This work shows that different variations during the microorganism growth can be detected by the sensors and highlights which of these changes are indicative of biofilm formation. TSM sensors have been used to monitor in real time the development of *Staphylococcus epidermidis* and *Escherichia coli* biofilms, formed on the gold electrode of the quartz crystal resonators, without any coating. Strains with different ability to produce biofilm have been tested. It was shown that, once a first homogeneous adhesion of bacteria was produced on the substrate, the biofilm can be considered as a semi-infinite layer and the quartz sensor reflects only the viscoelastic properties of the region immediately adjacent to the resonator, not being sensitive to upper layers of the biofilm. The experiments allow the microrheological evaluation of the complex shear modulus (G* = *G*′ + *jG*″) of the biofilm at 5 MHz and at 15 MHz, showing that the characteristic parameter that indicates the adhesion of a biofilm for the case of *S. epidermidis* and *E. coli*, is an increase in the resonance frequency shift of the quartz crystal sensor, which is connected with an increase of the real shear modulus, related to the elasticity or stiffness of the layer. In addition both the real and the imaginary shear modulus are frequency dependent at these high frequencies in biofilms.

## 1. Introduction

Biofilms are biological communities formed by microorganisms irreversibly adhered to a substrate, which are surrounded by a self-produced matrix based on polymeric substances [1]. These so called Extracellular Polymeric Substances (EPS) are mainly composed by polysaccharides, proteins, nucleic acids and lipids [1,2]. Among other things the matrix serves as a bonding mechanism. Biofilms have great relevance in clinical settings because they are associated with infections in medical implants [3]. In the case of biofilms in catheters, matrix stability determines biofilm detachment and the size of the resulting embolus [1]. Biofilms can be found in all media containing bacteria, and it is considered that under appropriate environmental conditions, most microorganisms are capable of forming them [4].

Biofilm formation provides bacteria with protection from environmental hazards of a physical and chemical nature. This fact explains the higher resistance of infections growing under biofilm type structures to the action of antibiotics, or the strength of fouling colonies attached to submerged surfaces. Within the properties of a biofilm, its viscoelastic characteristics play a relevant role for the success of this protective structure. Following [5], very little is known about the mechanical characteristics of biofilms, although these properties are of primary importance to explain the tenacity of biofilms associated with solid surfaces, their sessile survival in flowing environments (like rivers, oceans or blood flow), the shear-mediated dynamic behavior related with the colony spreading, or their resistance to mechanical removal strategies. Their mechanical stability is closely related to the exopolysaccharides present in the matrix [1]. Furthermore, it is known that the chemical resistance of biofilms is also related to their structure, and therefore, to their mechanical properties. Penetration of antimicrobials and nutrients into a biofilm depends on the degree of channelization and the presence of a suitable medium for molecular transport through the biofilm [6].

The most commonly applied methods to study biofilms are destructive, requiring the removal of the biofilm from the substrate [7] to analyze its chemical and biological composition. In a different way, continuous non-destructive monitoring techniques allow a dynamic analysis of biofilms (formation, evolution, destruction) and can be applied to analyze the response of the biofilm to changing conditions. Microscopic, spectrochemical, electrochemical, rheological and piezoelectric methods have been used to obtain information using non-invasive and online approaches [7,8]. Among them, both rheological and piezoelectric methods are well suited to provide information about the mechanical characteristics of biofilms. Quartz crystal sensors used in the present work are framed within the piezoelectric methods. 

Different experimental approaches like AFM [9], particle tracking [10], and rheometry [11,12] have been used to characterize the viscoelastic properties of biofilms and the interactions between cells and surfaces. Rheometers are able to provide information about the relations between stresses and strains in biofilms for different excitation conditions. TSM resonators can also be used to characterize the viscoelastic properties of media when these media are excited using a high frequency oscillating stress (typically 5–20 MHz). Although viscoelastic parameters have no linear dependency with frequency, there is a close relation between the measurements obtained by the rheometer and TSM resonators as shown by Castro [13]. The use of such resonators for the mechanical characterization of biofilms shows some advantages in relation to rheometers: TSMs are small sensors, which can be placed inside a microbial culture, they can be easily implemented in multiple channel set-up or even be placed out of the lab in open environments, for example, to investigate underwater biofilms in their native ecosystem.

An AT-cut quartz crystal was firstly used as a sensor in the work of Sauerbrey in the 50 s [14]. Originally TSM sensors were simply applied to monitor the mass of thin layers of solids deposited on their surface and measured in vacuum [15]. Afterwards, the liquid as a medium in contact with the sensor began to be used [16,17]. Given the sensitivity obtained, TSM resonators were considered as useful bio-medical, physical and chemical sensors in several applications. As a result, TSM resonators have been used to measure the viscosity of fluids [18], elasticity [19], mass [20], thickness [21], rheological parameters [22], etc. They can be applied to a wide variety of loadings such as fine solid layers [23], gases [24], viscous liquids [25] or combination of layers with solid particles [26]. In this work, quartz crystals are used to measure the changes of the viscoelastic properties and to obtain rheological parameters of biofilms during their evolution in a non-destructive setting.

While many works have shown that TSM sensors are capable of detecting cells and biofilm growth [27,28,29], analyses of the viscoelastic properties of biofilms during their formation at different frequencies (MHz regime) are still scarce and rarely performed. As stated before, these properties are a key factor to understand the communal lifecycles of bacteria, as they measure how they bind together and dissociate [10]. Shear modulus of elasticity (or rigidity constant) and shear viscosity are critical parameters influencing biofilm characteristics such as the biofilm hydration and fluidity which drives nutrient interchange and dissemination, or the EPS cohesion, which is directly related to biofilm fouling properties [30,31,32]. Marinkova and co-authors [33], studied the biofilms formed by *E. coli* and *P. fluorescens* during 5–17 h in several gel matrices. In this work, variations of 100 kPa–300 kPa of the real and imaginary parts of the shear modulus at 6 MHz were reported. Caplain and co-authors [34], analyzed the real and the imaginary shear modulus of a biofilm formed by *S. cerevisiae* during 80 h over a TSM resonator with a fundamental frequency of 5 MHz. Li and co-authors analyzed the viscoelastic properties of MC3T3 fibroblast monolayer by TSM quartz resonators [35]. In these works, the complex shear modulus was studied at the sensor fundamental frequency.

Nevertheless there are some aspects which were not discussed in detail in the aforementioned works about quartz sensors and biofilms. The density and viscosity of a liquid culture medium change when a microorganism grows in it, due to the cell metabolism and the increased population of cells. This phenomenon causes changes in the resonant properties of the sensor, i.e., both the resonant frequency shift and the dissipation factor. Moreover, gravitational precipitation of debris from dead bacteria that fall on the sensor surface and the weak adhesion of such substances to this surface may occur. These phenomena also cause shifts in the resonant properties of quartz [36,37]. As a result, not all the electrical variations registered by the resonator are related exclusively to biofilm formation. All these concurrent phenomena often make it difficult to differentiate between the onset of biofilm formation and other changes associated to microbial growth such as changes of the culture medium or cell deposition on the sensor. The aim of this work is to analyze and discuss them to consistently relate the measurements obtained by TSM resonators with the biofilm shear modulus.

To that end, TSM resonators were used to monitor the microbial growth and biofilm formation of *S. epidermidis* in a liquid culture medium (Tryptic Soy Broth—TSB). This study was performed in real time and non-destructively. Two strains of *S. epidermidis* (which differ in their ability to produce biofilms) have been continuously monitored using piezoelectric TSM resonators, at 37 °C in the selected culture medium. The use of the same bacteria but with different biofilm-producing ability allows discriminating between the changes in the resonant behavior of the sensor produced by the biofilm formation and the changes produced by other factors like microbial metabolism and non-attached bacteria deposition. Additionally, a strain of *E. coli* (which also produces biofilm) was monitored, in this case at 30 °C in another liquid culture medium (Colony Forming Antigen—CFA). Similarities and differences of biofilms formed by *E. coli* and *S. epidermidis* are discussed. Microbial growth and biofilm formation was followed by measuring changes in the resonance properties of the sensor (both the resonance frequency and dissipation factor) at the fundamental and the third harmonic. Ultrasonic mechanical parameters (the real and the imaginary part of the shear modulus) of the *S. epidermidis* and the *E. coli* biofilms developed on the sensor surface, are obtained for both frequencies. This analysis provides information about the frequency dependence of these parameters (real and imaginary part) at the high frequency regime.

## 2. Materials and Methods

### 2.1. Measurement Setup

**Figure 1:**
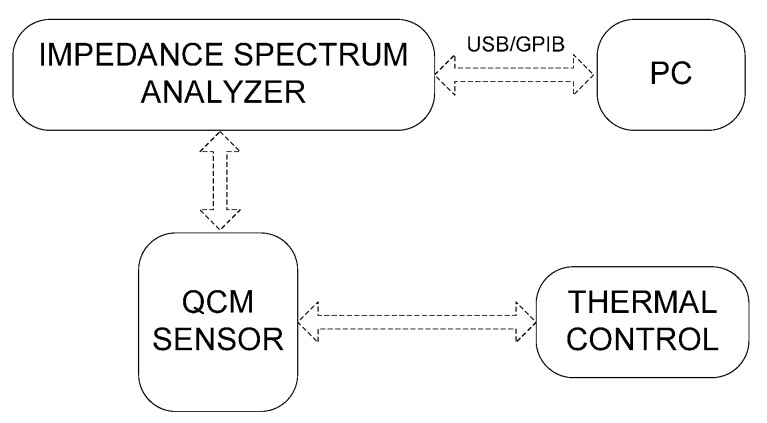
Experimental setup.

Figure 1 shows the experimental setup. AT-cut quartz crystals with a fundamental frequency of 5 MHz, 25.4 mm total diameter, 12.6 mm electrode diameter and polished surface finish were used (Stanford Research Systems, Sunnyvale, CA, USA). The quartz crystals were used with a 0100RH TSM support device with a 0100FC flow cell (*Stanford Research Systems*). It has a cavity of 0.6 mL over the crystal quartz. The input and output of the flow cell were connected by a flexible hose (with a volume of 1.2 mL). The measuring cell has O-rings on both the top and the bottom of the crystal quartz, to ensure the water tightness of the cavities. Once the liquid culture medium with suspended bacteria was introduced, the hydraulic circuit was closed and the flow stopped, to ensure the stability of the measurements.

**Figure 2:**
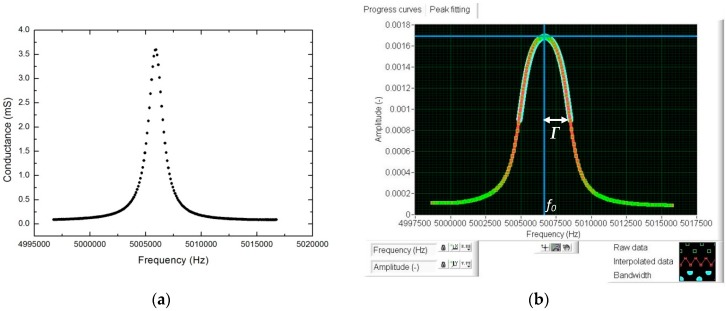
Example of acquisition of the real part of the admittance for the fundamental frequency of 5 MHz (**a**,**b**) the calculation of the values of the resonant frequency and the bandwidth using interpolation.

The crystal quartz was connected to an Agilent 4294A Precision Impedance Analyzer (Agilent Technologies, Santa Clara, CA, USA) ranging from 40 Hz to 110 MHz. The real part of the admittance (Figure 2a) was acquired around the resonance (fundamental and third harmonic) using a span of 20 kHz after performing an average of 20 signals. This spectrum was digitalized in 801 points. To improve the resolution, a cubic interpolation with a frequency step of 0.05 Hz, was used in the bandwidth region (see Figure 2b). The resonance frequency (*f*_0_) was determined as the peak maximum conductance (real part of the admittance), which corresponds to the series resonant frequency of the equivalent circuit Butterworth-van Dyke [38,39]. The bandwidth (2*Γ*) was calculated as the difference between the two frequencies at which the amplitude of the conductance equals the maximum value divided by two.

### 2.2. Microbial Cultures

Biofilms formed by two different bacteria, *S. epidermidis* and *E. coli* have been chosen for the tests. Both are easily found in the environment, and form a robust biofilm suitable for experimentation, since they cause enough changes in the resonant properties of the sensor.

*S. epidermidis* is a Gram-positive bacteria with spherical morphology and 0.5–1 μm diameter. Standard bacterial strains of *S. epidermidis* were obtained from the Servicio de Microbiología Clínica of the Hospital Central de la Defensa Gómez-Ulla (Madrid, Spain). Two strains of *S. epidermidis* have been monitored, which differ in their ability to produce EPS. The *ATCC 35984* strain is a high biofilm producer and, on the contrary, the *ATCC 12228* strain do not secrete EPS [40]. The lack of EPS, however, does not prevent certain adhesion and accumulation of bacteria and lysis on the gold electrode of the quartz crystal sensor. Therefore, different mechanical properties (adhesiveness, stiffness, etc.) of the material in contact to the gold electrode are expected during the growth of both strains.

Pure bacterial strain cultures of *S. epidermidis* were inoculated on Columbia agar platescontaining 5% sheep blood (Francisco Soria Melguiros S.A., Madrid, Spain) and introduced into a 311DS incubator (Labnet International Inc., Edison, NJ, USA) at a controlled temperature of 37 °C for a minimum of 24 h (and a maximum of 72 h). After that period, the bacteria were introduced into the liquid culture medium TSB (Francisco Soria Melguiros S.A.) until the cell suspension is adjusted to a concentration of 1.5 × 10^8^ cells/mL. This concentration is determined by measuring the optical density at 600 nm (OD_600_) using a UVmini-1240 UV-visible spectrophotometer (Shimadzu, Sidney, Australia).

*E. coli* is a Gram-negative bacterium with a slightly elongated shape of about 2 μm of length and 0.5 μm of wide. The bacterial strain *E. coli TRGM* (MG1655) was provided by the Biología Medioambiental group of the Centro de Investigaciones Biológicas (CIB, CSIC, Madrid, Spain). It was grown in the CFA culture medium [41]. For the tests, pure bacterial strain cultures of *E. coli* were grown in that medium at 30 °C for 1 day (using the 311DS shaking incubator set at 150 rpm). In this case, the cell suspension is adjusted to a concentration of 1 × 10^7^ cells/mL, by measuring OD_600_. It should be noted that initial concentration of bacteria for the case of *S. epidermidis* and *E. coli* are different. In both cases, concentrations were selected so that the biofilm formation and the changes in the resonance parameters happen in a few hours.

### 2.3. Experimental Procedure

The measuring cell is sealed and submerged in a FP45H thermal bath (Julabo, Seelbach, Germany) which controls the temperature with an accuracy of ±0.01 °C, since the sensor is sensitive to temperature changes. Temperatures of 37.00 °C and 30.00 °C was established for testing *S. epidermidis* and *E. coli*, respectively. The LabVIEW virtual programming software (National Instruments Corporation, Austin, TX, USA) was used to record the output signals and to visualize the results. Before the inoculum was made, the measuring cell and the culture medium were thermostated to minimize the time required to reach the appropriate temperature (around 30 min), and thus prevent thermal changes which mask the effect of biofilm development on the quartz sensor. Once the liquid culture medium with the bacteria is introduced into the measuring cell, the monitoring process starts. The interval between successive acquisitions was 60 s.

### 2.4. Biofilm Formation

**Figure 3:**
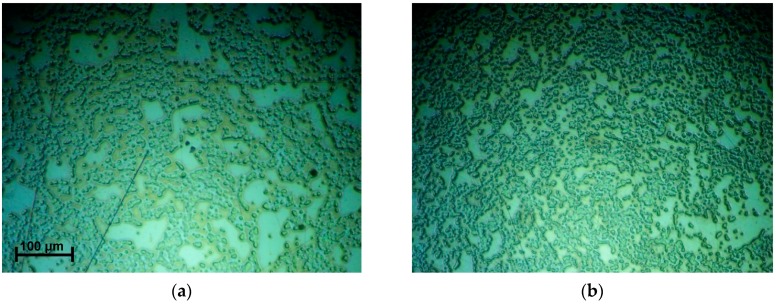

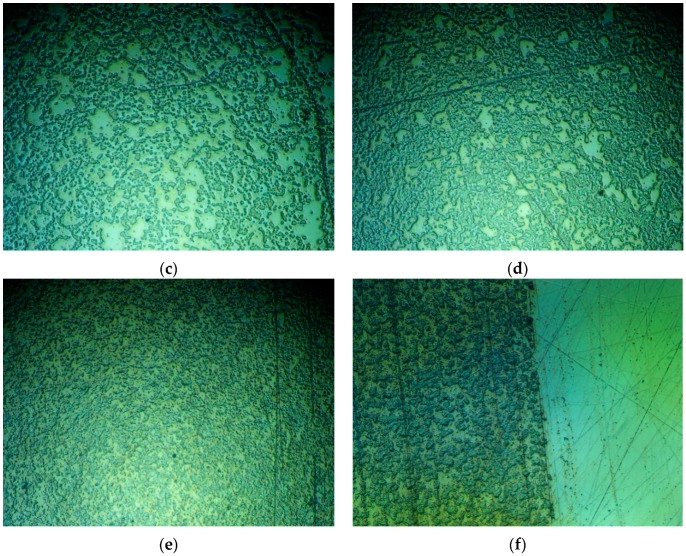
Time evolution of the biofilm produced by *S. epidermidis ATCC 35984* on the gold electrode of the AT-cut crystal sensor. (**a**) 45 min; (**b**) 90 min; (**c**) 180 min; (**d**) 330 min; (**e**) 24 h; (**f**) 24 h with a cleaned area on the right.

An Eclipse 50i optical microscope (*Nikon*, Tokyo, Japan) was used to observe biofilm development in its different phases. In Figure 3, the evolution of images captured after different biofilm formation periods is shown.

All the samples shown in Figure 3 were obtained with the sensor placed in a horizontal position during the course of biofilm formation. There is a significant deposition of cells surrounded by biofilm even from the first capture (45 min). As time increases, the biofilm created on the gold electrode becomes more uniform and more populated. In Figure 3f, a portion of the crystal was cleaned by wiping gently with a piece of paper with ethanol to compare the clean surface of the electrode (after 24 h) to the area covered by the biofilm. To obtain all the images shown in Figure 3, it was necessary to interrupt the formation of biofilm. Therefore, each image corresponds to a different microbiological growing experiment, always made under the same conditions.

### 2.5. Acoustical Parameters

The evolution of the dissipation factor and the resonance frequency of the fundamental and third harmonic of the TSM sensor during the microbial growth were registered. The dissipation factor is defined as the inverse of the quality factor:

(1) $$D=\frac{2\Gamma }{f_{0}},$$

where *Γ* is the half of the bandwidth and *f*_0_ is the resonant frequency (see Figure 2b).

To represent the results, the dissipation factor (Δ*D*) and the resonance frequency shift (Δ*f*_0_), are referred to the initial instant. For an infinite viscoelastic layer in contact to an ideal sensor, the complex shear modulus (G*) can be determined at the respective frequency by [42]:

(2) $$G^{*}\left(\omega \right)\rho =-{\left(\pi Z_{q}\frac{\Delta f^{*}}{f_{0}}\right)}^{2}$$

where Δf* is the complex resonance frequency shift (Δf* = Δ*f* + *j*Δ*Γ*) of the resonator referred to the unloaded measurement. *Z_q_* is the acoustic impedance of the quartz crystal sensor, *ρ* is the density of the sample. As will be shown in Section 3.2, biofilm is seen by our sensor as an infinite viscoelastic layer. *G*′ is the storage modulus and *G*″ is the loss modulus. The last equation can be rewritten as:

(3) $$G^{*}\left(\omega \right)\rho =-C^{*}{\left(\Delta f^{*}\right)}^{2}$$

where C* is a constant which depends theoretically on the characteristics of the quartz crystal. Nevertheless, for a real sensor, such a constant depends also on variables which are difficult to determine theoretically: crystal binding to the holder, holder geometry, etc., and this makes this constant complex, having both real and imaginary components. This constant is experimentally obtained by previously made calibration measurements. If a Newtonian liquid is used for this calibration, *G*′ is null and *G*″ = *ηω*, where *η* is the viscosity of the liquid and *ω* is the angular frequency. Once C* is known, the product of the density by the rheological parameters *G*′ and *G*″ of the viscoelastic medium contacting the sensor can be obtained. The shear modulus dependence with the frequency has no general equation for a non-Newtonian fluid or viscoelastic medium. Some works describe the dependence by a power-law exponent which may be equal or not for both the real and imaginary coefficients [35,43,44]. This dependence is discussed in Section 3.2.

## 3. Results and Discussion

### 3.1. Evolution of the Electrical Resonance Parameters

**Figure 4:**
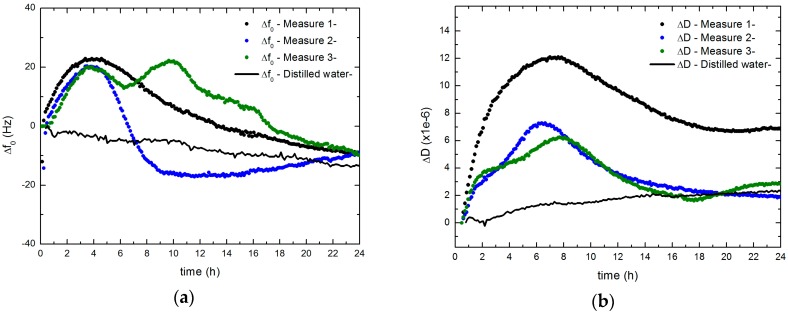
Biofilm formation processes of *S. epidermidis* ATCC 35984 (**a**) Δ*f*_0_ and (**b**) ∆*D* for 3 measurements made with the quartz resonator at the fundamental frequency of 5 MHz.

In this section, the resonance frequency shifts (Δ*f*_0_) and the dissipation factor changes (∆*D*) obtained along 24 h during the development of the *S. epidermidis* biofilm are analyzed. The initial instant corresponds to the bacterial inoculum. Figure 4 shows the results of three experiments developed under the same conditions, and the reference with distilled water at 37 °C. The biofilm formation processes present important differences among them, but all they follow similar steps. The variability of these measurements is related to the intrinsic random nature of the biofilm development and, in general, of any process of microbial growth. In the water test, after the first 40 min, both frequency and dissipation increments reach become stable and changes less than 0.5 Hz/h and 2.6 × 10^−5^/h respectively, are found. These slopes are clearly lower than those obtained during biofilm formation and may be attributed to instabilities in the acquisition electronics or in the liquid sample (for example, bubble trap while cell filling).

In all the cases tested, the biofilm formation was characterized by an initial increase of the resonance frequency that occurs during the first 4 h. This behavior coincides with an increase of the dissipation factor, which extends for 2–4 more hours before it begins to decline.

The positive shift in the resonance frequency has also been reported in other QCM studies with bacterial adhesion to the electrode [45,46,47]. An increasing of the dissipation factor also was detected [46,47]. The behavior of resonance parameters with biofilm formation agrees with those obtained by Chen et al. [45], by Olofsson et al. [46] for biofilms of *Bacillus cereus* and *Bacillus subtilis*, and by Reipa et al. [47] for biofilms of *Pseudomonas aeruginosa*.

Due to the measurement cell configuration, the microbial growth occurs with a limited presence of oxygen and without agitation. Therefore, although it is not a strictly anaerobic growth, presumably oxygen reserves become exhausted quickly during microbial growth (microaerophilic conditions). This is consistent with the fact that from the beginning of the test, there is a considerable variation of the resonance properties, which means that the biofilm formation starts early, as it was found for the case of anaerobic conditions for the *S. epidermidis* biofilm [48].

**Figure 5:**
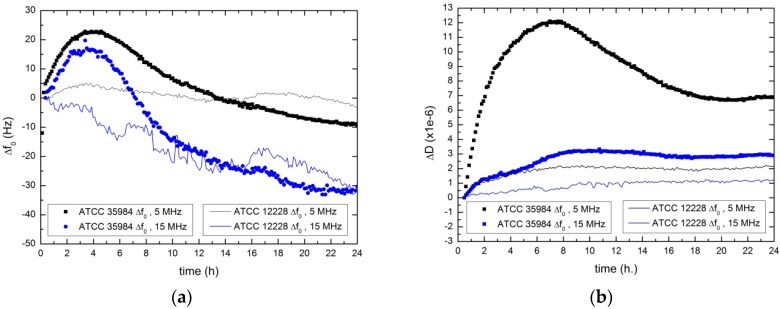
Evolution of Δ*f*_0_ (**a**) and ∆*D* parameter (**b**) for the two frequencies analyzed (5 MHz –black- and 15 MHz –blue-). The biofilm-producing (*ATCC 35984*) and non-producing (*ATCC 12228*) strain tests are plotted with symbols and continuous lines, respectively.

In Figure 5, the evolution of the electrical resonance parameters Δ*f*_0_ and ∆*D* for the case of the biofilm-producing strain (*ATCC 35984*, with symbols) and for non-producing strain (*ATCC 12228*, with solid line) are shown. The figure plots the measurements at the fundamental and the third harmonic (5 MHz –black symbols and lines- and 15 MHz –blue symbols and lines) of the sensor. In Figure 5a, resonance frequency shifts are plotted, while the dissipation factor shifts for the same microbial growth are displayed in Figure 5b.

The main difference between the results obtained for *ATCC 35984* and *ATCC 12228*, is the previously commented increase of the resonance frequency (in the case of non-producer strain this phenomenon was not detected). Furthermore, the change in slope of the Δ*D* parameter is significantly greater in the case of the producer strain compared to the non-producer strain.

It’s remarkable that non-biofilm producing strains also induce detectable changes in the resonance properties of the sensor. In this case, some cells were also deposited on the sensor surface, although they were not attached as tightly as in the case of the producer strain and EPS material were not present. This deposition can be related to the slight increment of the resonance frequency at the beginning of the experiment.

In addition, the sensor can be sensitive to the changes occurring in the environment due to metabolic processes; although there was no production of biofilm, bacteria multiplied within the liquid culture medium, taking and releasing nutrients and changing the properties of the medium in contact with the sensor. In the case of the producer strain, the biofilm formed a viscoelastic coating over the sensor. As the following section will show, once a certain biofilm thickness (on the order of hundreds of nanometers) is exceeded, this coating isolates the sensor from the medium beyond. In the case of the non-producer strain, this viscoelastic coating does not appear, and the sensor remains sensitive to changes that occur in the liquid culture medium (involving changes in metabolism and variations of density and viscosity). When this medium remains as a Newtonian fluid, the shifts of the dissipation factor and the resonance frequency are related [17]: a resonance frequency decreasing corresponds to a dissipation factor increasing and vice versa. This behavior should be shown by the non-producing strain in Figure 5. Nevertheless the resonance frequency at 5 MHz shows an almost steady behavior, probably due to the interference caused by the deposition of dead bacteria. This deposition does not attach the cells to the surface, which would result into a frequency increasing as it is observed for the biofilm producer strain.

In relation to the frequency, the behavior of Δ*f*_0_ and ∆*D* show a similar trend for 5 MHz and 15 MHz in Figure 5. As it was described for the 5 MHz curve, the 15 MHz Δ*f*_0_ curve increases during the first hours as a result of the biofilm adhesion. For both frequencies the difference among the producing and non-producing strain at the maximum frequency increment reaches 20 Hz approximately. The dissipation factor also exhibits an increasing trend at 15 MHz, although the changes are less pronounced for this third harmonic.

### 3.2. Evolution of the Biofilm Mechanical Parameters

Changes in the resonance properties of the quartz resonator TSM seen in Section 3.1 are mostly caused by the presence of the EPS matrix self-produced by the bacterial cells, and the viscous connections among bacteria. They all form a viscoelastic layer on the gold electrode of the quartz crystal sensor. Following the results obtained by microscopy (Figure 3), the biofilm formed after the first hour can be considered uniform enough along the sensor surface to apply a one-dimensional model.

**Figure 6:**
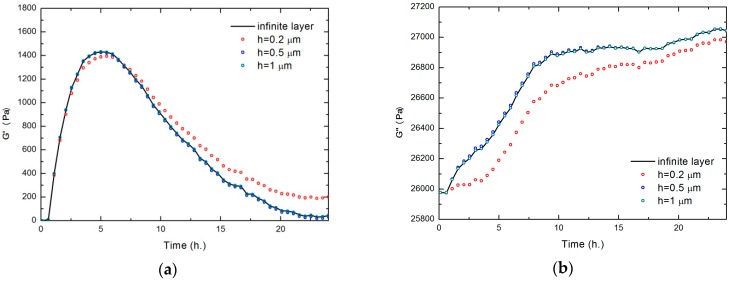
Influence of thickness, *h*, biofilm layer during its formation. Time curve of the real shear modulus, *G*′ (**a**) and the imaginary shear modulus; *G*″ (**b**) at the frequency of 5 MHz. *S. epidermidis* ATCC 35984 producing strain.

According to the methodology detailed in Section 2.5, we can obtain the rheological parameters, the real and imaginary part of the shear modulus, at various frequencies, if we have a semi-infinite viscoelastic layer. However, for a biofilm thickness of the order of the wavelength (a few hundred of nanometers) resonance effects within the biofilm layer may influence Δ*f*_0_ and Δ*D* parameters [49]. In Figure 6 this effect is analyzed from the changes in Δ*f*_0_ and Δ*D* shown in Figure 5. Making use of the transmission-line model for TSM quartz resonator sensors [50] and assuming a constant thickness biofilm layer over the sensor surface, the rigidity constant could be estimated. This circuit is valid to perform a one-dimensional analysis of a piezoelectric resonator with a layer surrounded by a viscoelastic medium (in our case, the culture medium). A transmission line is used to model the biofilm (with a constant thickness and complex shear modulus). The parameters used for the simulations are written in Table 1. Thus, in Figure 6 the stiffness constant of the material is estimated along the biofilm formation process, for different thicknesses of biofilm (0.2 μm, 0.5 μm, 1 μm and infinite).

It can be seen that, for layer thicknesses greater than 0.5 microns, there is no influence of the interface between the biofilm and the culture medium because no detectable shear wave could be reflected from such interface. As a result, when the biofilm thickness grows beyond this range, the changes detected on the rigidity constant cannot be related to the finite layer reflections and, therefore, they only can be related to rigidity changes experienced by the biofilm directly in contact to the surface.

**Figure 7:**
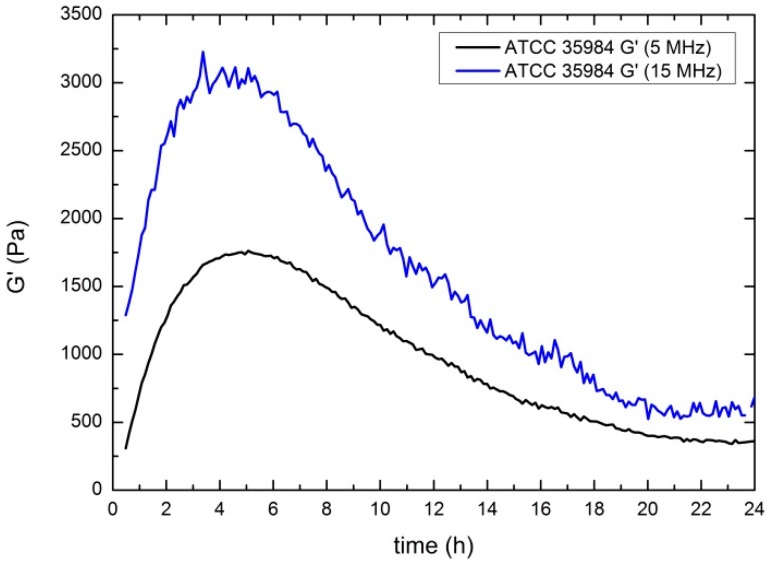
Evolution of the real shear modulus for 5 MHz (black) and 15 MHz (blue). Results obtained for *S. epidermidis* ATCC 35984 (biofilm producing) strain.

As a conclusion, the results shown in Figure 6, and microscopy images (shown in Figure 3), suggest that one hour after the inoculation (when the layer can be considered as uniform along the surface), the biofilm can be modeled as an infinite layer adhered to the sensor. Thus, from this time, the sensor detects only viscoelastic (both the real shear modulus and the imaginary shear modulus) changes in the vicinity of the sensor, not being sensitive to the thickness of the layer. In Figure 7, the results corresponding to the real shear modulus obtained at 5 MHz and 15 MHz are shown.

**Figure 8:**
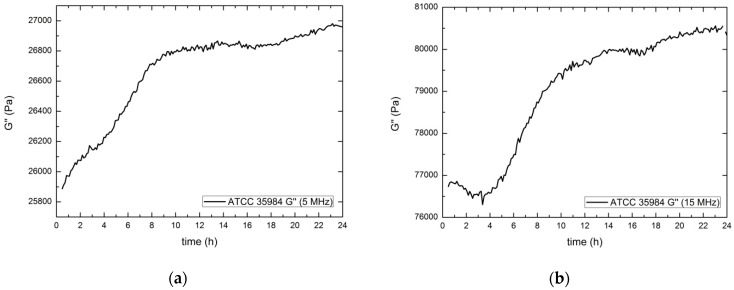
Evolution of the imaginary shear modulus for 5 MHz (**a**) and 15 MHz (**b**). Results obtained for *S. epidermidis* ATCC 35984 producer strain.

The main feature is the significant increase in *G*′ values, reaching a maximum after the first 4–5 h. The following decrease of *G*′ values detected could be compatible with a loss of rigidity probably caused by the consumption of some of the nutrients present in the EPS by bacteria, by the increasing acidity of the growth medium [12] or by some other metabolic result. In Figure 8, the results corresponding for the imaginary shear modulus both 5 MHz (a) and 15 MHz (b) are shown.

Following Figure 8, after 8 h, the measured value is almost stabilized, increasing with a small slope related to an increase of the biofilm viscosity, probably caused by the presence of extracellular *teichoic* acid [51]. This behavior with two slopes resembles that obtained for *S. epidermidis* at the same temperature by Gutiérrez and co-authors [52], using an electric impedance-based method (real time cell analyzer—RTCA). The electrical impedance is related to the mobility of ions in contact with the electrodes. Taking into account that this mobility may be directly related to the medium viscosity through the imaginary part of the rigidity constant, the analogy obtained between both measurements (electrical impedance and *G*″) is consistent.

To calculate *G*′ and *G*″ using expression (2) the density of the biofilm was considered constant, and its value assumed to be of the same order as the cell contents. Cells are composed essentially of water (70–80%) and a set of macromolecules (polysaccharides, proteins, lipids and nucleotides) plus a small concentration of salts and ions. Although the actual value of the cell composition (and the EPS produced) will depend on the kind of cell, following [53], a value of 1100 kg/m^3^ biofilm density (*ρ*) was assumed. This value was estimated for the yeast *S. cerevisiae* and taking into account that there are great similarities in the cellular content of diverse microorganisms, which is mainly water, a great deviation from the exact density of the *S. epidermidis* biofilm is not expected.

According to the rheological results obtained, the values of *G*″ exceed those of *G*′ (both at 5 MHz and at 15 MHz), that is, the viscous contribution dominates over the elastic contribution on the rigidity constant of the biofilm formed by bacteria *S. epidermidis*. This was not found at low frequencies; Pavlovsky and co-authors [11], measured *G*′ and *G*″ parameters of the biofilm formed during the first 7 h by *S. epidermidis* in TSB culture medium with a shear rheometer, using oscillations in the range (0.01–10 Hz). At a frequency of 1 Hz, values of (11 ± 3) Pa and (1.9 ± 0.5) Pa respectively were found by them. In some studies dealing with cells [35,43,44], the dependence of the complex shear modulus (G^*^(*ω*)) with the frequency is described as:

(4) $$G^{*}\left(\omega \right)={\bar{G}}_{0}\left(1+j\eta \right){\left(\frac{\omega }{\omega_{0}}\right)}^{\alpha }+j\omega \mu$$

where *η* is the hysteresivity or structural damping coefficient of the model, *α* is the power-law exponent, ${\bar{G}}_{0}$ and *ω*_0_ are scale factors for the moduli and frequency respectively. From this model, the storage modulus follows a power-law with an exponent *α*, and the loss modulus has the same power-law dependence but with an additional Newtonian viscous term. However, it is noteworthy that if α ≤ 1, the trend of data shows a higher increasing of *G*″ with frequency. Therefore even if *G*′ > *G*″ at low frequencies, *G*″ would surpass the *G*′ value at a given frequency. In Section 3.2 this frequency dependence is calculated for both *S. epidermidis* and *E. coli*.

Ruhs and co-authors [12] measured the rheological parameters of biofilms formed by various bacterial strains for long times (50–120 h) under different conditions (pH, temperature, culture medium) using interfacial rheometry. They show that the real shear modulus, for the specific case of *E. coli* and *P. fluorescens* growing in LB medium, experience local maxima as it was found in this work, which were related to metabolic causes (depletion of glucose and increasing acidity of the medium which diminished elasticity).

Reports of rheological parameters obtained using TSM resonators during the formation of biofilms are still scarce. Marinkova and co-authors [33] analyzed the biofilm of *E. coli* and *P. fluorescens* formed during 5–17 h in various gel matrices. These matrices favor a variation up to 100 kPa and 300 kPa of *G*′ and *G*″, respectively, at 6 MHz, respectively, much higher than ours (which were one order of magnitude lower for *G*″, and almost two to *G*′, for 5 MHz), presenting in both cases values that increase with time. No local maxima were found, but fresh medium was added during the measurements, which avoids nutrient depletion or other physical-chemical changes in the medium. Caplain and co-authors [34] measured the values of *G*′ and *G*″ for a biofilm formed by *S. cerevisiae* in YPD medium under aerobic conditions during 80 h, by a TSM resonator with a fundamental frequency of 5 MHz. The experimental conditions are closer to those used in the present work (homogeneous liquid culture medium, no fresh medium added). A series of oscillations of the rigidity constants appeared in their results, which were related to self-organizing life cycles of the strain. The paper shows that the imaginary shear modulus increases over time, regardless of the appearance of oscillations, and the real shear modulus increase up to 60 h when the trend is reversed. The values obtained are close to 10 kPa for *G*′ and 40 kPa for the case of *G*″, which are in the order of magnitude obtained in this work.

### 3.3. Characterization of the E. coli Biofilm

**Figure 9:**
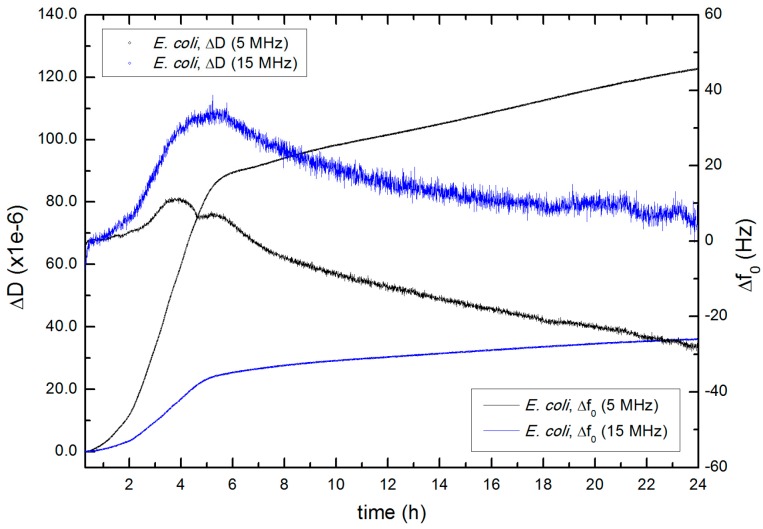
Evolution of ∆*D* and Δ*f*_0_ (5 MHz and 15 MHz) for *E. coli* TRGM (MG1655) biofilm formation. Measurement made in CFA at 30 °C.

The changes of the TSM sensor resonance properties obtained during the biofilm formation of *E. coli* TRGM (MG1655) are similar than those shown previously for *S. epidermidis*. In Figure 9 the Δ*D* and Δ*f*_0_ results are shown (at 5 MHz and at 15 MHz).

As it was found for the case of *S. epidermidis*, ∆*D* begins to increase until it reached a slight and linear increasing trend 5 h after the bacteria was inoculated. The Δ*f*_0_ parameter presents an initial increasing behavior, with a maximum at −5 h, approximately. After this maximum the frequency values slowly decreased.

**Figure 10:**
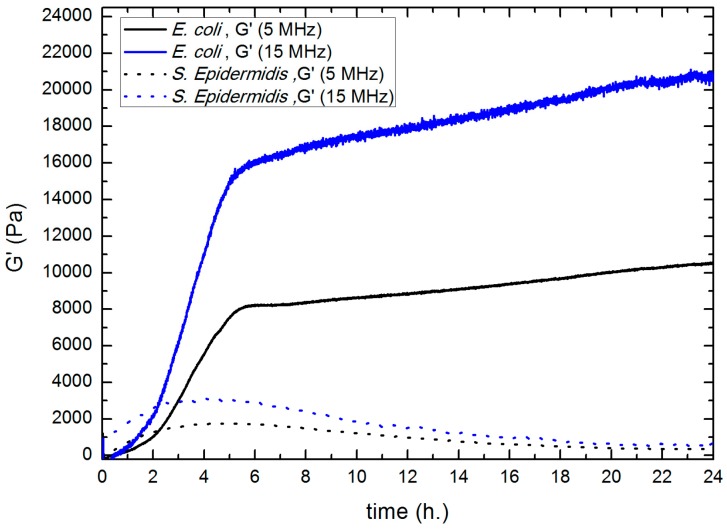
Evolution of the real shear modulus for 5 MHz (black) and 15 MHz (blue). Biofilm formed by *S. epidermidis* and *E. coli* are represented by dot line and solid line, respectively.

Values of the real shear modulus were calculated and compared to *S. epidermidis* biofilm at 5 MHz and 15 MHz (Figure 10). This curve shows that the biofilm formed by the *E. coli* strain is much stiffer than that formed by *S. epidermidis*, being its *G*′ values higher from the first 2 h of biofilm formation. The *G*′ values of *E. coli* and *S. epidermidis* reach 8200 Pa and 1750 Pa respectively, at 5 MHz, and 16,000 Pa and 3000 Pa at 15 MHz. The decreasing trend of this parameter found in the case of *S. epidermidis*, does not appear for *E. coli*, at least during the first 24 h of culture.

**Figure 11:**
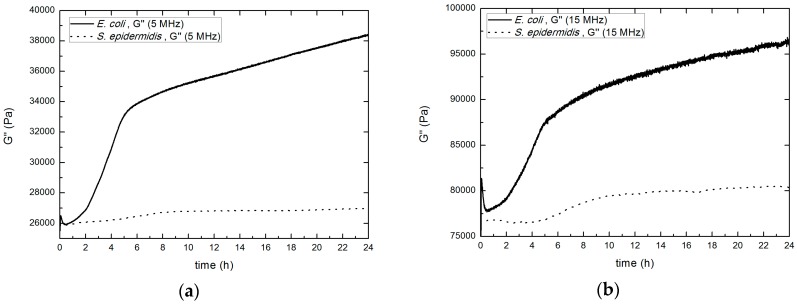
Evolution of the imaginary shear modulus for 5 MHz (**a**) and 15 MHz (**b**). Biofilm formed by *S. epidermidis* and *E. coli* are represented by dot line and solid line, respectively.

In Figure 11, values of the imaginary shear modulus are shown, both at 5 MHz (a) and at 15 MHz (b). The viscosity of the biofilm formed by *E. coli* is much higher than the *S. epidermidis* biofilm. Therefore, it’s clear than the biofilm formed by *E. coli* is more tightly adhered to the surface by both viscous and rigid mechanisms than the biofilm formed by *S. epidermidis*. This tighter adhesion makes the cell spreading more difficult for the *E. coli* than for *S. epidermidis*; on the contrary, the *E. coli* biofilm will be more resistant to mechanical antimicrobial actions.

Values of *G*′ and *G*″ for the *E. coli* biofilm are close to those obtained by Caplain and co-authors [34] for the biofilm caused by *S. cerevisiae*, which also reached approximately 10 and 40 kPa, respectively after 24 h.

**Figure 12:**
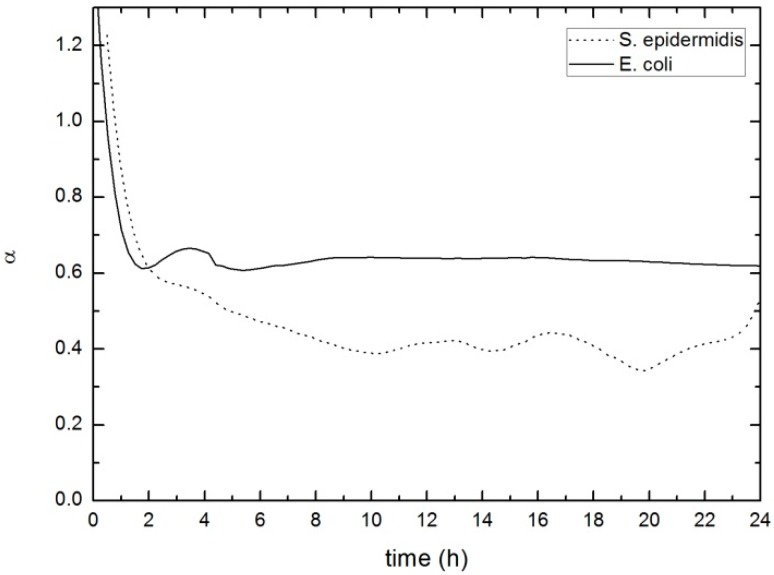
Evolution of the power-law exponent –*α-* for the biofilm formed by *S. epidermidis* (dot line) and by *E. coli* (solid line).

Following Equation (4), and our measurements at 5 MHz and 15 MHz, we can obtain the values of the power-law exponent, α, for both biofilms (*S. epidermidis* and *E. coli*). These values are shown in the Figure 12. It can be seen that once the biofilm begins to attach to the crystal surface, this exponent begins to stabilize, remaining fairly constant from the two first hours of measurement with no further significant variations. References do not clarify the physical significance of *α*, however the behavior obtained in these tests may suggest that the nature of the material forming the biofilm remains stable even when the rigidity constant suffers changes along time.

The *α* value obtained for the biofilm within this work is in the order of 0.4–0.6, which is somewhat upper than those shown in studies considering cells, but no EPS matrix (0.15–0.35) [35,43,44]. This fact reflects the relevance of the EPS matrix over the structural characteristics of the biofilm. Further theoretical and experimental studies about the frequency related behavior of biofilms are needed to provide a deeper knowledge about the role of this parameter and its relation with the physical properties of the biofilm.

## 4. Conclusions

There is a great interest in the characterization of biofilm mechanical properties due to the relevant role they play in the bacterial survival capacity through the bacterial spreading ability, the biofilm resistance to mechanical removal strategies, the mechanical stability and the chemical resistance, which is directly related to the antimicrobial efficiency. Previous works have already shown that TSM resonators constitute a powerful tool for the analysis of biofilms. They are compact and can be implemented as a non-destructive technique for biofilm formation monitoring, keeping the biofilm intact during the measurement. The technique has a high sensitivity allowing the detection of biofilms from the beginning. Nevertheless there are only a few works discussing the viscoelastic properties of biofilms measured using such resonators.

In this paper, different phenomena causing changes on the resonance parameters of TSM sensors during the bacterial and biofilm growth were presented. It was shown that from the early stages of biofilm formation (one hour after inoculation for the experiments presented), the biofilm can be considered as a semi-infinite viscoelastic layer for the TSM sensor. Therefore, the resonator is sensitive just to the viscoelastic properties of the layer immediately adjacent to its surface, being blind to what happens a few hundred nanometers beyond the sensor surface.

It was shown that the most distinctive feature of the emergence of a biofilm is the increase in the real shear modulus (related to the elasticity or stiffness of the layer). Regarding the behavior of other parameters such as the imaginary shear modulus, we can conclude that changes in the imaginary shear modulus alone are not exclusive of biofilm formation, since similar variations caused by strains which do not produce biofilm were registered. These variations are related to both the change of the properties of the medium by the metabolic action of the cells during the growth and the deposition of cell debris. In terms of the electrical resonance parameters, biofilm formation is characterized by the increase of the resonance frequency of the sensor, which, in the literature, is associated with the adhesion of bacteria to the substrate [46,47]. This behavior was shown in this paper for two different bacteria *S. epidermidis* (strain ATCC 35984), Gram-positive, and *E. coli* (MG1655), Gram-negative.

It was shown that for biofilms both the real shear modulus (*G*′) and the imaginary shear modulus (*G*″) are frequency dependent at these high frequencies. Nevertheless, a more complete theoretical and experimental study is required to fully describe the behavior of these dependence of the complex shear modulus (G*(*ω*)) with the frequency. All these results point out the great potential of TSM sensors as a tester for biofilm related processes, for example, to analyze the effectiveness of inhibitors, although changes not strictly related to the biofilm, like cell deposition or liquid medium composition, has to be also considered to obtain consistent conclusions from the experiments.

## Figures and Tables

**Table 1:** Density and viscosity of the fluids in contact with the sensor at 37 °C.

	Culture Medium	Air
**Density, ρ** (kg/m^3^)	1030	1.2
**Viscosity, η** (Pa.s)	0.88 × 10^−3^	1.8 × 10^−5^
